# Evaluation of the nutrition literacy assessment questionnaire for college students and identification of the influencing factors of their nutrition literacy

**DOI:** 10.1186/s12889-023-17062-z

**Published:** 2023-10-30

**Authors:** Wang Yan, Hu Caihong, Yang Xuefeng, Zhang Jiayu

**Affiliations:** 1 Hospital of China University of Geosciences (Wuhan), Wuhan, Hubei 430074 China; 2 School of Public Health, Tongji Medical College, Huazhong University of Science and Technology, Wuhan, China; 3 School of Medicine and Health Management, Tongji Medical College, Huazhong University of Science and Technology, No. 13 Hangkong Road, Qiaokou Region, Wuhan City, Hubei Province China

**Keywords:** College students, Nutrition literacy, Reliability evaluation, Validity evaluation, Influencing factors

## Abstract

**Background:**

Nutrition health has become a major public health issue in both high and middle-income countries. Nutrition literacy is an important indicator to evaluate the effect of public health intervention and one of the important concepts in health promotion. Thus, this study aimed to verify the reliability and validity of a nutrition literacy assessment questionnaire (NLAQ) and investigate the associated factors of nutrition literacy among college students.

**Methods:**

We conducted a cross-sectional online survey of college students from April to November 2022 in Wuhan (*N* = 774). We employed the Cronbach’s alpha coefficient, exploratory and confirmatory factor analysis to evaluate the reliability and validity. We used latent profile analysis to classify the nutrition literacy. We conducted Chi-square test and binary logistic regression to identify the influencing factors.

**Results:**

The Cronbach’s alpha coefficient of the NLAQ and its dimension was ranging from 0.837 to 0.909. The common factors were consistent with the original dimensions. All indicators met the requirements (χ^2^/df = 6.16 < 8, GFI = 0.929, NFI = 0.939, CFI = 0.948, RMSEA = 0.082 < 0.1). College students’ disciplines (*χ*^*2*^ = 7.769, *P* = 0.021), mothers’ education level (*χ*^*2*^ = 26.599, *P* < 0.001), and fathers’ occupation type (*χ*^*2*^ = 11.218, *P* = 0.024) had impacts on nutrition literacy.

**Conclusion:**

The NLAQ has good reliability and validity, and could be used as a measurement tool to evaluate college students’ nutrition literacy. Schools and families should take targeted measures to improve the college students’ nutrition literacy.

**Supplementary Information:**

The online version contains supplementary material available at 10.1186/s12889-023-17062-z.

## Introduction

Nutrition literacy is understood as a ‘specific form of health literacy’, and defined as the individual capacity to obtain, process, and understand basic nutrition information for making appropriate nutrition decisions [1–3]. Given the central role of nutrition in health and chronic disease prevention, nutrition literacy is an evaluation indicator for the effectiveness of public health, health education, and health promotion work [4]. College students are in a period of transition to independent living, and may easily develop unhealthy eating habits because of pressures such as new environments, learning environments or family environments [5]. Therefore, improving the nutrition literacy of college students is of great significance to improve their dietary nutrition, enhance their physical quality, and even improve the national health level.

Different researchers have developed various nutrition literacy measurement tools according to different types of populations. According to Nutbeam’s concept of health literacy, nutrition literacy is categorized into three forms: functional, interactive, and critical [4, 6]. Most authors emphasized practical knowledge and skills to regulate food intake and the importance of attitudes, awareness, motivation, or concrete behavior to act on knowledge and skills [7–12]. These tools all focused on the elements of functional health literacy [13]. Other researchers also developed critical nutrition literacy scales from the interactive and critical view. These scales highlighted interactive competencies to share information and transfer skills, the ability to critically judge the quality of nutrition information, and the capacity to recognize the effect of food and nutrition decisions on society [10, 14, 15]. However, few domestic nutrition literacy scales were designed in relation to college students. Wang et al. developed a nutrition literacy scale through expert consultation based on the relevant foreign scales from the functional and interactive perspectives [16]. Mo et al. conceptualized and validated a short-form nutrition literacy assessment tool for Chinese college students based on a 43-item nutrition literacy measurement scale [17].

Numerous factors significantly affect the health literacy level among college students. Researchers in different countries using the health literacy questionnaire have confirmed that sociodemographic variables (e.g., age, gender), learning characteristics (e.g., year, field of study), family environment (e.g., parents’ education, socioeconomic status, area of residence of the family, employment status) could affect health literacy [18–22]. Meanwhile, comparable findings were reported in studies conducted domestically using various health literacy scales [23–26]. However, few studies were conducted on influencing factors of nutrition literacy among college students. Lai et al. confirmed that factors associated with healthy eating behavior could enhance nutrition literacy [5]. Students who were from urban areas, living with both parents, and with high academic performance were more likely to report higher nutrition literacy levels [27].

Dietary nutrition is increasingly recognized as a major public health concern in China, particularly among college students owing to the rapid population growth of highly educated citizens. Foreign researchers have also developed different nutrition literacy assessment instruments for various occupational groups, and explored the impact of college students’ sociodemographic characteristics on health literacy around the world. Therefore, strengthening the verification and influencing factors of nutrition literacy among college students in China is urgently needed. The nutrition literacy assessment questionnaire (NLAQ) is the first tool that specially designed for Chinese college students to assess their nutrition literacy level [16]. However, the application of NLAQ still needs to be widely validated in other regions. Thus, this study aimed to (1) examine the reliability and validity of the NLAQ for future practical application and (2) explore factors that influence the level of nutrition literacy among college students for enhancing their health quality.

## Methods

### Sample size calculation

The pre-survey results showed that the nutrition literacy level was 35.0%. The sample size was calculated by the formula: *n* = t^2^pq/d^2^ = 743 (Assuming α = 0.05, t = 2, p = 0.35, q = 1-p = 0.65, d = 0.1 × p = 0.035) [28]. This sample size was considered sufficient to identify risk factors in multivariate analysis.

### Sampling survey

**Fig. 1:**
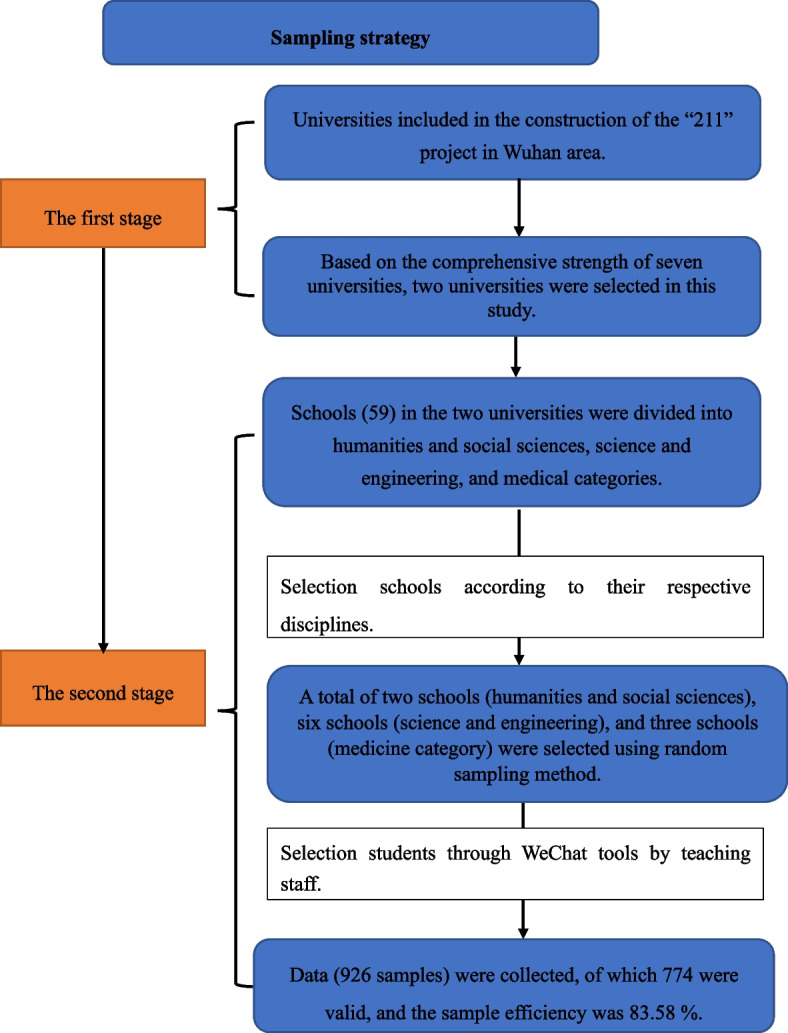
Flowchart of the participants selection from the “211” project universities in Wuhan area

The present study was an observational cross-sectional study. We conducted this survey in Wuhan, which is the capital of Hubei Province, and locates in Central China. The city has more than 80 universities with a total of 1.683 million college students, which is comparatively higher than other provincial capitals in China. However, only seven universities in Wuhan were included in the construction of the “211” project, which was officially launched in 1995 with the approval of the State Council. We collected data using two-stage stratified cluster random sampling in seven “211” project universities. At the first stage, we selected two universities based on the comprehensive strength of seven universities. The first university covers 11 subject categories, which include disciplines related to nutritional literacy, while the second university covers only 8 subject categories. At the second stage, we stratified 59 schools of the two universities into humanities and social sciences, science and engineering, and medical categories based on their respective disciplines. Then, we selected two schools from the humanities and social sciences, six schools from the science and engineering, and three schools from the medicine category for random sampling based on the proportion of disciplines in universities. The staff from teaching offices in 11 departments used WeChat tools to distribute the questionnaire via the Wenjuanxing platform from April to November 2022 and reminded them to participate in this survey voluntarily. A total of 926 samples were collected, of which 774 were valid, and the sample effective rate was 83.58%. The flowchart in Fig. 1 provides a visual display of the sampling strategy.

### Design

The self-administered questionnaire was divided into two parts.

#### Part 1 different characteristics

Part 1 consisted of general personal, academic, and family environment characteristics. General personal characteristics include gender, nationality, age, and living costs (in CNY). Academic characteristics include online learning time, disciplines, and academic performance. Family environment characteristics include residential area, average annual household income (in CNY), parents’ education level and parents’ occupation type.

#### Part 2 nutrition literacy assessment questionnaire

The NLAQ was developed by Chinese researchers on the basis of stage-specific “critical nutrition literacy” scales [10, 16, 29]. The questionnaire consists of 13 items covering three domains of nutrition literacy: obtaining information (four items), understanding information (six items), and evaluation and application of information (three items). Items were scored on a five-point Likert scale ranging from one (totally disagree) to five (totally agree). The specific items of the scale were listed in Supplementary file 1.

### Data analysis

The SPSS (version 21.0), AMOS (version 19.0), and MPLUS (version 8.3) software were used for statistical analysis. Quantitative data were expressed as mean and standard deviation. Qualitative data were expressed as frequency and percentage. Cronbach’s alpha coefficient and item-total correlation coefficient were applied to measure the reliability analysis. Construct validity was assessed through exploratory and confirmatory factor analyses. Standardized factor loading (λ), measurement error (θ), average variance extracted (AVE), and construct reliability (CR) were used to measure convergent validity. Latent profile analysis (LPA) was employed to determine the category of nutrition literacy among college students. Chi-square test and binary logistic regression were utilized for univariate and multivariate analysis, respectively. *P* < 0.05 indicated a statistically significant difference.

Several fit indices such as the Akaike information criterion (AIC), Bayesian information criterion (BIC), adjusted-Akaike information criterion (ABIC), bootstrap likelihood ratio test (BLRT), Lo–Mendell–Rubin test (LMRT) and entropy were selected as the basis for classification in LPA [30]. Generally, the lower the value of AIC, BIC, and ABIC, the better the fitting degree of the sample data model. Additionally, a higher entropy value indicated a relatively better fit, with values ≥ 0.8 indicating well-separated profiles [31]. Besides, P value of LMRT and BLRT that were lower than 0.05 indicated that the model fit of the k categories was better than that of the k-1 categories [32]. In addition, the corresponding profile prevalence was generally required to be no less than 5%. Taken together, these findings suggest that the number of latent profiles was determined by a combination of indicators and the practical significance of different categories.

A KMO value higher than 0.7 was considered to be acceptable for factor analysis according to Kaiser’s point of view [33]. The following indicators were selected as the criteria for judging whether or not the model was suitable for confirmatory factor analysis (Supplementary file 2).

## Results

### Reliability analysis

An overall Cronbach’s alpha coefficient of 0.909 was obtained from the reliability analysis. In the questionnaire, internal consistency was determined for obtaining, understanding, and evaluation and application of dietary nutrition information on the questionnaire with Cronbach’s alpha values of 0.887, 0.899, and 0.837, respectively. These values were all above 0.8, suggesting that the instrument had good reliability at these three levels [34]. The item-total correlations coefficients among the three levels on the questionnaire were all higher than 0.75 (Table 1).

**Table 1:** The reliability test of nutrition literacy assessment questionnaire among college students

First-level indicator	Second-level indicator	Item-total correlation coefficients	Alpha if Item Deleted	Cronbach’s alpha
Obtaining information	Q1_1	0.876	0.848	0.887
	Q1_2	0.909	0.824	
	Q1_3	0.860	0.857	
	Q1_4	0.810	0.884	
Understanding information	Q2_1	0.803	0.883	0.899
	Q2_2	0.808	0.882	
	Q2_3	0.856	0.873	
	Q2_4	0.837	0.878	
	Q2_5	0.803	0.883	
	Q2_6	0.788	0.888	
Evaluation and application information	Q3_1	0.867	0.787	0.837
	Q3_2	0.879	0.749	
	Q3_3	0.860	0.785	

### Validity analysis

#### Construct validity

Exploratory factor analysis showed that the KMO, χ^2^, and *P* values were 0.9, 6233.912, and < 0.001, with a cumulative variance contribution rate of 71.878%. The factor loadings of each item were consistent with the theoretical assumptions after the oblique rotation. Factors 1, 2, and 3 correspond to understanding information, obtaining information, and the evaluation and application information. Further, the factor loading of each item ranged from 0.667 to 0.880 (Supplementary file 3). Confirmatory factor analysis revealed that the values of χ^2^ / df, GFI, NFI, CFI, and RMSEA were 6.16, 0.9, 0.939, 0.948, and 0.082, respectively. All these indicators met the statistical requirements.

#### Convergent validity

The convergent validity test of the NLAQ among college students showed that the λ values were greater than 0.7 except for Q1_4 (0.694) and the θ values were lower than 0.5 except for Q1_4 (0.518). The AVE and CR of each indicator were greater than 0.6 and lower than 0.8, indicating that the questionnaire had good convergence validity. All potential variables at different levels passed the convergence validity test. The λ, θ, AVE, and CR values are shown in Table 2.

**Table 2:** The convergent validity test of nutrition literacy assessment questionnaire and nutrition literacy level among college students

Indicator	λ	θ	AVE	CR	Mean	Standard Deviation
Nutrition literacy			0.619	0.950	3.17	0.65
Obtaining information			0.668	0.888	2.93	0.84
Q1_1	0.858	0.264				
Q1_2	0.917	0.159				
Q1_3	0.783	0.387				
Q1_4	0.694	0.518				
Understanding information			0.603	0.901	3.29	0.73
Q2_1	0.758	0.425				
Q2_2	0.749	0.439				
Q2_3	0.829	0.313				
Q2_4	0.800	0.360				
Q2_5	0.772	0.404				
Q2_6	0.747	0.442				
Evaluation and application information			0.627	0.834	3.26	0.81
Q3_1	0.810	0.344				
Q3_2	0.830	0.311				
Q3_3	0.731	0.466				

### Subjects characteristics

Table 3 presents the different characteristics of the included students. Regarding the personal characteristics, of the 774 participants, 57.5% were male, 89% were of Han nationality, and their mean age was mostly concentrated between 18–23 years old. For their academic characteristics, the participants consisted of 518 college students with online learning time > 3 h, accounting for 66.93% of the sample. Most students were sophomores (51.7%) and science and engineering students (49.6%). In terms of family environment characteristics, students with residences in county-level cities and average annual household income of 50,000–100,000 CNY accounted for 28.04% and 37.08% of participants, respectively. The parents’ education levels and occupational types of the respondents were similar, and most of them were had education of junior high school and below and were engaged in individual businesses or employed as general staff. The detailed data of other features are shown in Table 3.

**Table 3:** The results of univariate analysis on college students’ nutrition literacy assessment questionnaire

Variable	Items	N (%)	Weak nutrition literacy	Strong nutrition literacy	χ^2^	*P*
Gender	Male	445 (57.5)	288 (58.1)	157 (56.5)	0.184	0.668
	Female	329 (42.5)	208 (41.9)	121 (43.5)		
Nationality	Han	689 (89.0)	438 (88.3)	251 (90.3)	0.715	0.398
	Minority	85 (11.0)	58 (11.7)	27 (9.7)		
Age	≤ 20	706 (91.2)	453 (91.3)	253 (91)	0.023	0.879
	> 20	68 (8.8)	43 (8.7)	25 (9)		
Living costs (CNY)	≤ 1000	98 (12.7)	68 (13.7)	30 (10.8)	3.718	0.156
	1000–2000	544 (70.3)	352 (71)	192 (69.1)		
	> 2000	132 (17.1)	76 (15.3)	56 (20.1)		
Grade	First	113 (14.6)	68 (13.7)	45 (16.2)	6.089	0.193
	Second	400 (51.7)	272 (54.8)	128 (46)		
	Third	147 (19.0)	89 (17.9)	58 (20.9)		
	Forth	76 (9.8)	43 (8.7)	33 (11.9)		
	Fifth	38 (4.9)	24 (4.8)	14 (5)		
Online learning time	< 1h	26 (3.4)	18 (3.6)	8 (2.9)	5.262	0.154
	1–2h	77 (10.0)	51 (10.3)	26 (9.4)		
	2–3h	153 (19.8)	86 (17.3)	67 (24.1)		
	> 3h	518 (66.9)	341 (68.8)	177 (63.7)		
Disciplines	Humanities and social sciences	140 (18.1)	103 (20.8)	37 (13.3)	11.511	0.003
	Science and engineering	384 (49.6)	251 (50.6)	133 (47.8)		
	Medicine	250 (32.3)	142 (28.6)	108 (38.8)		
Academic performance	20% and below	207 (26.7)	130 (26.2)	77 (27.7)	9.175	0.057
	20–40%	187 (24.2)	116 (23.4)	71 (25.5)		
	40–60%	211 (27.3)	127 (25.6)	84 (30.2)		
	60–80%	99 (12.8)	68 (13.7)	31 (11.2)		
	80% and above	70 (9.0)	55 (11.1)	15 (5.4)		
Residential area	Municipalities/provincial capitals	148 (19.1)	79 (15.9)	69 (24.8)	16.198	0.003
	Prefecture-level city	163 (21.1)	97 (19.6)	66 (23.7)		
	County-level cities	217 (28.0)	147 (29.6)	70 (25.2)		
	Town	78 (10.1)	50 (10.1)	28 (10.1)		
	Rural areas	168 (21.7)	123 (24.8)	45 (16.2)		
Average annual household income (CNY)	50,000 and below	197 (25.5)	141 (28.4)	56 (20.1)	10.411	0.015
	50,000–100,000	287 (37.1)	186 (37.5)	101 (36.3)		
	100,000–200,000	195 (25.2)	118 (23.8)	77 (27.7)		
	200,000 and above	95 (12.3)	51 (10.3)	44 (15.8)		
Fathers’ education level	Junior high school and below	294 (38.0)	216 (43.5)	78 (28.1)	24.752	< 0.001
	Senior high school	207 (26.7)	134 (27)	73 (26.3)		
	Technical secondary school /junior college	96 (12.4)	50 (10.1)	46 (16.5)		
	Undergraduate and above	177 (22.9)	96 (19.4)	81 (29.1)		
Mothers’ education level	Junior high school and below	372 (48.1)	269 (54.2)	103 (37.1)	33.052	< 0.001
	Senior high school	172 (22.2)	109 (22)	63 (22.7)		
	Technical secondary school /junior college	102 (13.2)	61 (12.3)	41 (14.7)		
	Undergraduate and above	128 (16.5)	57 (11.5)	71 (25.5)		
Fathers’ occupation type	Unemployed, semi-unemployed or agricultural workers	141 (18.2)	103 (20.8)	38 (13.7)	13.676	0.008
	Workers or business service personnel	219 (28.3)	130 (26.2)	89 (32)		
	Individual businesses or general staff	259 (33.5)	176 (35.5)	83 (29.9)		
	Professional and technical personnel or private business owners	74 (9.6)	44 (8.9)	30 (10.8)		
	Senior managers or government leaders	81 (10.5)	43 (8.7)	38 (13.7)		
Mothers’ occupation type	Unemployed, semi-unemployed or agricultural workers	229 (29.6)	167 (33.7)	62 (22.3)	13.095	0.011
	Workers or business service personnel	205 (26.5)	128 (25.8)	77 (27.7)		
	Individual businesses or general staff	244 (31.5)	149 (30)	95 (34.2)		
	Professional and technical personnel or private business owners	55 (7.1)	30 (6)	25 (9)		
	Senior managers or government leaders	41 (5.3)	22 (4.4)	19 (6.8)		

### Nutrition literacy level and classification

**Fig. 2:**
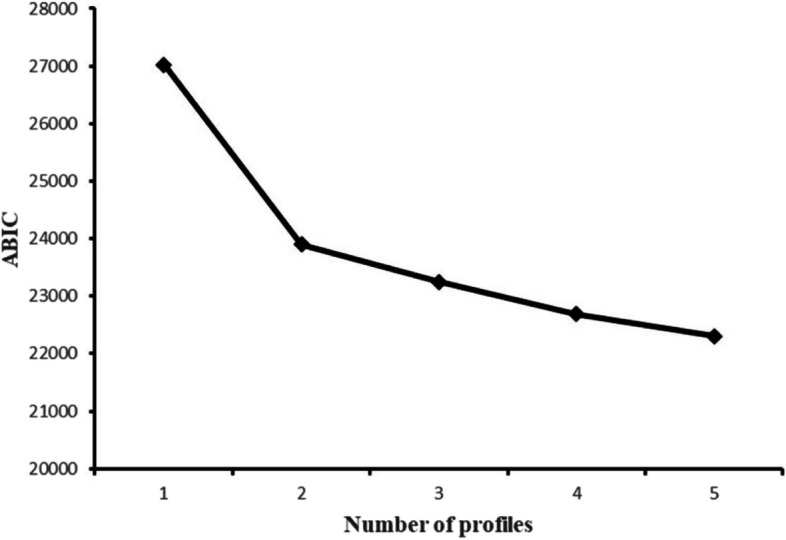
Scree plot of ABIC from LPA analysis based on nutrition literacy assessment questionnaire

**Fig. 3:**
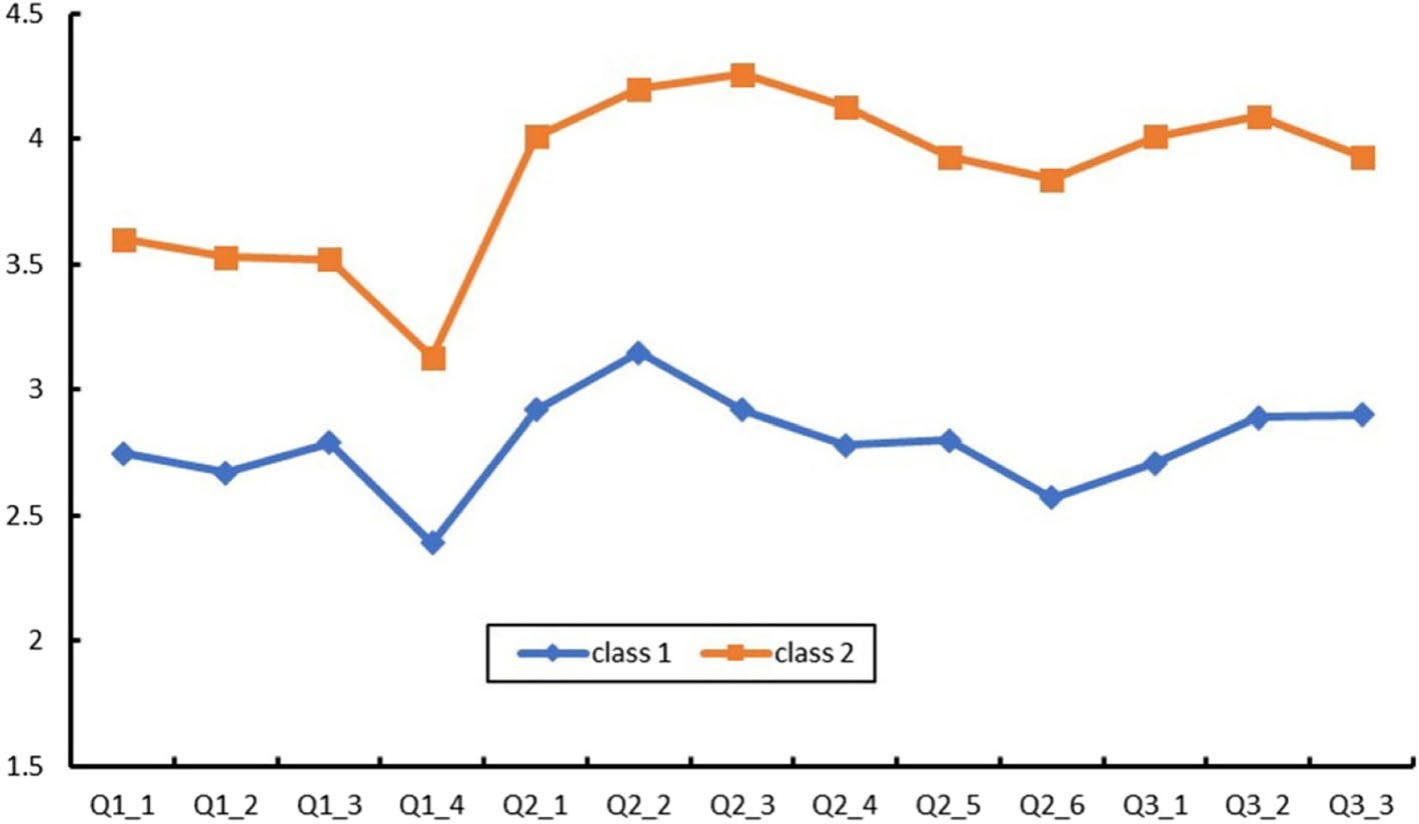
Three profiles of the best-fitting three-class pattern based on nutrition literacy assessment questionnaire 13 items

Table 2 indicates that the scores of the total dimension and the sub-dimension did not exceed 3.5 points. This study used LPA to optimally classify the level of nutrition literacy. The LPA results showed that the models converged in up to five profiles. The fit indices for the one- to five-profile solutions (Table 4) were subsequently compared. First, we observed that the value of entropy in each model was above 0.90, except for the four-profile solution (0.881); thus, all models could provide high classification accuracy. Second, the BLRT and LMRT results were significant for every model comparison, and these two indicators were non-informative for the current model selection. Third, as the number of profiles increased, the AIC, BIC and ABIC values continued to decrease across the five profiles. Fourth, the proportion of Model 5 after classification was 4%, which failed to meet the minimum requirement of 5%. Fifth, we found an “elbow” point at the two-profile solution from the scree plot, which indicated a considerably improved fit when the number of latent profiles increased from 1 to 2 (Fig. 2). The proportions of students in Model 2 were 35.8% and 64.2%. Moreover, the scores of each item in Group 1 were lower than those in Group 2, with statistically significant differences (Fig. 3). Therefore, Group 1 was regarded as the “weak nutrition literacy” group and Group 2 as the “strong nutrition literacy” counterpart.

**Table 4:** Model fit indices for one- to five-latent profile solutions and corresponding profile prevalence

Model	AIC	BIC	ABIC	Entropy	LMRT *P*	BLRT *P*	Profile prevalence
1	26,982.683	27,103.624	27,021.062				
2	23,841.99	24,028.062	23,901.043	0.936	< 0.001	< 0.001	0.642/0.358
3	23,168.347	23,419.532	23,248.056	0.926	0.0015	< 0.001	0.587/0.185/0.228
4	22,585.052	22,901.359	22,685.427	0.881	0.0039	< 0.001	0.155/0.378/0.239/0.228
5	22,183.736	22,565.165	22,304.776	0.901	0.0048	< 0.001	0.23/0.133/0.379/0.218/0.04

### Analysis of differences in variables according to different characteristics

Univariate analysis revealed that general personal characteristics (specifically, gender, nationality, and age) had no effect on the dietary health literacy of college students, but academic characteristics (specifically, discipline) and family characteristics such as family address, family income, parents’ education level and occupation type had different nutrition literacy levels (Table 3).

All the significant predictors of nutrition literacy in the univariate analysis were incorporated into the binary logistic regression model, and the results are presented in Table 5. Multivariate analysis revealed that disciplines, mothers’ education level, and fathers’ occupation type showed significant results in the final model. Medical students (vs humanities and social science students, OR [95%CI] = 1.475[0.946–2.301]), mothers with higher education level, and the father’s occupation type for workers or business service personnel (vs unemployed, semi-unemployed or agricultural workers, OR [95%CI] = 1.714[1.072–2.740]) were more likely to belong to the strong nutrition literacy group.

**Table 5:** The result of binary logistic regression analysis on college students’ nutrition literacy

Variables	β	SE	χ^2^	*P*	OR (95%CI)
**Disciplines**			7.769	0.021	
Humanities and social sciences^a^					1
Science and engineering	0.389	0.227	2.934	0.087	1.475 (0.946–2.301)
Medicine	0.657	0.238	7.619	0.006	1.929 (1.210–3.076)
**Mothers’ education level**			26.599	< 0.001	
Junior high school and below^a^					1
Senior high school	0.486	0.207	5.509	0.019	1.625 (1.083–2.438)
Technical secondary school /junior college	0.732	0.258	8.017	0.005	2.078 (1.253–3.449)
Undergraduate and above	1.294	0.254	25.987	< 0.001	3.646 (2.217–5.995)
**Fathers’ occupation type**			11.218	0.024	
Unemployed, semi-unemployed or agricultural workers^a^					1
Workers or business service personnel	0.539	0.240	5.056	0.025	1.714 (1.072–2.740)
Individual businesses or general staff	-0.099	0.251	0.155	0.694	0.906 (0.554–1.483)
Professional and technical personnel or private business owners	-0.061	0.340	0.032	0.857	0.941 (0.483–1.830)
Senior managers or government leaders	0.094	0.335	0.078	0.780	1.098 (0.569–2.120)
**Constant**	-1.564	0.273	32.811	< 0.001	

^a^Note the reference group

## Discussion

By confirming the reliability and validity of the NLAQ, and exploring factors that influenced college students’ nutrition literacy, our study revealed that 1) the NLAQ had good reliability and validity, and 2) academic characteristics and family environment-related factors could promote the nutrition literacy level of students.

### Reliability and validity of the NLAQ

Reliability analysis reflects the stability of a scale’s structure [35]. This study used Cronbach’s alpha coefficient and item-total correlation to evaluate the scale’s reliability. The alpha coefficient of the three dimensions of nutrition literacy was above 0.8, and the alpha coefficient of the scale was above 0.9, thus, the scale had good reliability. The item-score correlation coefficients were all higher than 0.50, indicating good internal consistency of the NLAQ. These outcomes were in accordance with the results of Wang et al., which confirmed that the alpha coefficient of each dimension was between 0.708 and 0.814 [16].

Effectiveness refers to the degree to which the instrument being tested corresponds precisely to the reality [36]. This study used construct and content validity to evaluate the scale’s validity. Exploratory factor analysis extracted three factors that explain 71.878% of the total data variance. Factor loadings for the 13 items were greater than 0.5, which met the requirements. The common factors were also consistent with the dimensions of the theoretical hypothesis. In addition, the fitting indices of structural validity reached acceptable standards, suggesting that the construction validity of the scale was suitable.

### Nutrition literacy level and classification

The score for nutrition literacy level did not exceed 3.5 points. The main reason for the low level of overall nutrition literacy may be caused by the acquisition of nutrition information. The channels for obtaining nutritional information in this scale focused on new media, ignoring the important fact that college students could also obtain nutritional information from the classroom or other channels. This work also used LPA to identify distinct classes of nutrition literacy levels among Chinese college students. The optimal model was determined through comparison of several fit indices and visual inspection of the scree plot. All fitting indices of the two-profile model all met the criteria, which indicated the model had strong discrimination.

### Factors associated with nutrition literacy

The nutrition literacy level of medical students was higher than that of non-medical students, an outcome which was consistent with most domestic studies on health literacy [26, 37, 38]. Various health literacy courses were naturally set up at medical colleges, a condition which could help medical students gain access to more dietary information. The nutrition literacy level of the participants was closely related to the family environment, such as family location and income, parental education level, and mother’s occupation type [39–42]. A positive correlation was found between family location and family income. Families located in rural areas and with lower incomes might pay more attention to basic living needs, while ignoring to increase nutrition literacy level. Further, family economy and parental occupation type were positively correlated with parental education level. The higher the education level of parents, the more opportunities and ways to learn health-related knowledge to improve their health, and the stronger the ability to obtain and understand nutrition information. Therefore, families with high education levels were more likely to create a healthy family atmosphere and provide guidance to their children about how to obtain, understand, evaluate, and apply nutrition information.

### Limitations

This study had some limitations. First, sample bias may exist in this work because the sample was limited to two universities in the Wuhan area. Second, the NLAQ used in this research was designed from functional perspective, continued exploration and verification is necessary using the scales designed from other perspectives. Third, the cross-sectional design of this research did not produce very precise or convincing results and generated uncertainty regarding the outcomes.

## Conclusion

This study confirmed that the NLAQ has good reliability and validity could be used as a measurement tool to evaluate college students’ nutrition literacy. Academic characteristics including disciplines and family environment characteristics including the mothers’ education level and fathers’ occupation type were significant predictors of nutrition literacy. Moreover, society and universities should pay greater attention to students with these characteristics, and take targeted measures to improve the nutrition literacy of college students. Further research is also needed to design more nutrition literacy scales aimed at other populations and explore other factors that influence the nutrition literacy levels.

### Supplementary Information

**Additional file 1.** Fit indicator criteria in confirmatory factor analysis.

**Additional file 2.** The results of exploratory factor analysis of nutrition literacy assessment questionnaire among college students.

**Additional file 3.** Nutrition literacy assessment questionnaire among college students.

## Data Availability

The datasets used and/or analysed during the current study are available from the corresponding author on reasonable request.

## References

[1] Fonseca-Pedrero E, Ortuño-Sierra J, de Álbeniz AP, Muñiz J, Cohen AS (2017). A latent profile analysis of schizotypal dimensions: associations with psychopathology and personality. Psychiatry Res.

[2] Blitstein JL, Evans WD (2006). Use of nutrition facts panels among adults who make household food purchasing decisions. J Nutr Educ Behav.

[3] Council NR (2004). Health literacy: a prescription to end confusion.

[4] Nishida C, Uauy R, Kumanyika S, Shetty P (2004). The joint WHO/FAO expert consultation on diet, nutrition and the prevention of chronic diseases: process, product and policy implications. Public Health Nutr.

[5] Lai IJ, Chang LC, Lee CK, Liao LL (2021). Nutrition literacy mediates the relationships between multi-level factors and college students’ healthy eating behavior: evidence from a cross-sectional study. Nutrients.

[6] Smith SK, Nutbeam D, McCaffery KJ (2013). Insights into the concept and measurement of health literacy from a study of shared decision-making in a low literacy population. J Health Psychol.

[7] Block LG, Grier SA, Childers TL, Davis B, Ebert JEJ, Kumanyika S, Laczniak RN, Machin JE, Motley CM, Peracchio L (2011). From nutrients to nurturance: a conceptual introduction to food well-being. J Public Policy Mark.

[8] Vidgen HA, Gallegos D (2014). Defining food literacy and its components. Appetite.

[9] Gibbs H, Chapman-Novakofski K (2013). Establishing content validity for the Nutrition Literacy Assessment Instrument. Prev Chronic Dis.

[10] Guttersrud O, Dalane J, Pettersen S (2014). Improving measurement in nutrition literacy research using Rasch modelling: examining construct validity of stage-specific ‘critical nutrition literacy’ scales. Public Health Nutr.

[11] Doustmohammadian A, Omidvar N, Keshavarz-Mohammadi N, Abdollahi M, Amini M, Eini-Zinab H (2017). Developing and validating a scale to measure Food and Nutrition Literacy (FNLIT) in elementary school children in Iran. PLoS One.

[12] Cassar AM, Denyer GS, O’Connor HT, Gifford JA (2018). A qualitative investigation to underpin the development of an electronic tool to assess nutrition literacy in Australians adults. Nutrients.

[13] Krause C, Sommerhalder K, Beer-Borst S, Abel T (2018). Just a subtle difference? Findings from a systematic review on definitions of nutrition literacy and food literacy. Health Promot Int.

[14] Slater J (2013). Is cooking dead? The state of Home Economics Food and Nutrition education in a Canadian province. Int J Consum Stud.

[15] Smith MG. Food or nutrition literacy? What concept should guide home economics education? International Federation of Home Economics; 2009.

[16] Wang JQ, Li F, Jia YN, Fu H (2021). Development of nutrition literacy assessment questionnaire for college students and the evaluation of reliability and validity. Fudan Univ J Med Sci.

[17] Mo G, Han S, Gao T, Sun Q, Zhang M, Liu H (2022). Development and validation of a novel short-form nutrition literacy measurement tool for Chinese college students. Front Public Health.

[18] Elsborg L, Krossdal F, Kayser L (2017). Health literacy among Danish university students enrolled in health-related study programmes. Scand J Public Health.

[19] Mackert M, Champlin S, Mabry-Flynn A (2017). Exploring college student health literacy: do methods of measurement matter?. J Student Aff Res Pract.

[20] Rababah JA, Al-Hammouri MM, Drew BL, Aldalaykeh M (2019). Health literacy: exploring disparities among college students. BMC Public Health.

[21] Zhang Y, Zhang F, Hu P, Huang W, Lu L, Bai R, Zhao Y (2016). Exploring health literacy in nursing students of Chongqing, China: a cross-sectional survey using the health literacy questionnaire. Lancet.

[22] Ayaz-Alkaya S, Terzi H (2019). Investigation of health literacy and affecting factors of nursing students. Nurse Educ Pract.

[23] Li X (2023). Health literacy and influencing factors of college students in Tianjin in 2021. Chin J Prev Contr Chron Dis.

[24] Liu RF, Zhang HM, Wang YL, Chen J, Hu QF (2023). Current situation of college students’ health literacy in Sichuan Province and influencing factors. Chin J PHM.

[25] Si JP, Wang XJ, Guo Q (2022). Research on the influencing factors and improvement paths of health literacy among college students in Henan Province. Soft Sci Health.

[26] Yang HJ, Zhang M, Wei QG, Zhou Y, Huang BH, Wang HX, Huang X (2022). Analysis on the status of health literacy and its influencing factors among college students in Guangzhou. Chin J Health Stat.

[27] Gao T, Duan Y, Qi Q, Mo G, Han S, Liu H, Zhang M (2023). Nutrition literacy differs based on demographics among university students in Bengbu, China. Front Public Health.

[28] Zhang Q, Hou XM, Yang YJ (2021). Dental erosion survey among 3–6 year old children in Xicheng district of Beijing. Contemp Med.

[29] Zhang L, Jia YN, Qian HH, Fu H (2018). Development of assessment indicators system of health literacy for college student in China. Health Educ Health Promot.

[30] Tein JY, Coxe S, Cham H (2013). Statistical power to detect the correct number of classes in latent profile analysis. Struct Equ Modeling.

[31] Li JB, Wu AMS, Feng LF, Deng Y, Li JH, Chen YX, Mai JC, Mo PKH, Lau JTF (2020). Classification of probable online social networking addiction: a latent profile analysis from a large-scale survey among Chinese adolescents. J Behav Addict.

[32] Kim SY (2014). Determining the number of latent classes in single- and multi-phase growth mixture models. Struct Equ Modeling.

[33] Stanley JC, Hopkins KD. Educational and psychological measurement and evaluation. Upper Saddle River: Prentice-Hall; 1972.

[34] Wu ML (2010). Questionnaire statistical analysis practice——SPSS operation and application.

[35] Koo TK, Li MY (2016). A guideline of selecting and reporting intraclass correlation coefficients for reliability research. J Chiropr Med.

[36] Cheng J, Coope C, Chai J, Oliver I, Kessel A, Wang D, Sun Y (2018). Knowledge and behaviors in relation to antibiotic use among rural residents in Anhui, China. Pharmacoepidemiol Drug Saf.

[37] Xu LN, Li HS, Wang Y, Bai LX, Yao Z, Cao WD, Xu H, Jiang C (2019). Analysis of the current situation and influencing factors of health literacy among college students in Beijing in 2016. Chin J Prev Contr Chron Dis.

[38] Guo YT, Fang C, Zhang ZX, Du DD, Chen J, Chen BH (2019). Survey on health literacy status among college students in Hubei Province. Chin J Health Educ.

[39] Sun T, Sun MW, Lian TY, Zhang Y, Ni JD (2019). Health literacy and its influencing factors among residents in community of Shenzhen. Chin J Soc Med.

[40] Pan XF, Ding Y, Hu YF, Chen RJ, Fan XY, Xia MK, Xu Y, Gu SB (2016). Research on health literacy evolution and correlative factors among residents of 15–69 years old in Shanghai during 2008–2015. Shanghai J Prev Med.

[41] Zhang L (2019). Current situation of health literacy of residents in China. Occup Health.

[42] Yuan XQ. Study on the status of health literacy and the relationship with health behavior and health status of undergraduates in two provinces. Master Thesis. Chinese Center for Disease Control and Prevention; 2019.

