# Supplementary material for: Evaluation of the nutrition literacy assessment questionnaire for college students and identification of the influencing factors of their nutrition literacy
Source: BMC Public Health. 2023 Oct 30;23:2127. doi: 10.1186/s12889-023-17062-z (PMC10617111; doi:10.1186/s12889-023-17062-z)
Supplement: Supplementary file 1 — Additional file 1. Fit indicator criteria in confirmatory factor analysis. [file 12889_2023_17062_MOESM1_ESM.docx]

Supplementary File 1 Fit indicator criteria in confirmatory factor analysis

| Absolute fit indicators | Value | relative fit indicators | Value |
| --- | --- | --- | --- |
| RMSEA | <0.1 | NFI | >0.9 |
| GFI | >0.8 | CFI | >0.9 |
| AGFI | >0.8 | TLI | >0.9 |
