# Supplementary material for: Evaluation of the nutrition literacy assessment questionnaire for college students and identification of the influencing factors of their nutrition literacy
Source: BMC Public Health. 2023 Oct 30;23:2127. doi: 10.1186/s12889-023-17062-z (PMC10617111; doi:10.1186/s12889-023-17062-z)
Supplement: Supplementary file 2 — Additional file 2. The results of exploratory factor analysis of nutrition literacy assessment questionnaire among college students. [file 12889_2023_17062_MOESM2_ESM.docx]

Supplementary File 2 The results of exploratory factor analysis of nutrition literacy assessment questionnaire among college students

| Item | Factor1 | Factor2 | Factor3 |
| --- | --- | --- | --- |
| Q2_4 | 0.825 |  |  |
| Q2_3 | 0.806 |  |  |
| Q2_2 | 0.751 |  |  |
| Q2_1 | 0.745 |  |  |
| Q2_5 | 0.733 |  |  |
| Q2_6 | 0.667 |  |  |
| Q1_2 |  | 0.880 |  |
| Q1_1 |  | 0.850 |  |
| Q1_3 |  | 0.830 |  |
| Q1_4 |  | 0.770 |  |
| Q3_3 |  |  | 0.841 |
| Q3_2 |  |  | 0.786 |
| Q3_1 |  |  | 0.680 |
