# Supplementary material for: Evaluation of the nutrition literacy assessment questionnaire for college students and identification of the influencing factors of their nutrition literacy
Source: BMC Public Health. 2023 Oct 30;23:2127. doi: 10.1186/s12889-023-17062-z (PMC10617111; doi:10.1186/s12889-023-17062-z)
Supplement: Supplementary file 3 — Additional file 3. Nutrition literacy assessment questionnaire among college students. [file 12889_2023_17062_MOESM3_ESM.docx]

**Nutrition literacy assessment questionnaire** **among college students**

**Obtaining information**

Q1-1 I am vulnerable to nutritional dietary advice on new media (Wechat, Weibo)

Q1-2 If I discuss food with others, I often refer to the information in the media

Q1-3 I mainly learn nutrition and diet knowledge from new media (Wechat, Weibo)

Q1-4 I believe in the various dietary suggestions I read in the media

**Understanding information**

Q2-1 I think the dietary guidelines are easy to understand

Q2-2 I understand the concept of “balanced diet”

Q2-3 I can understand the information on food labels (nutrients, energy, etc.)

Q2-4 When I read about nutrition and diet, I don’t need someone to help me understand it

Q2-5 I found the nutritionist’s statement easy to understand

Q2-6 I understand the core items and standards in the dietary guidelines

**Evaluation and application information**

Q3-1 I refer to the label information on the food package (nutrients, energy, etc.) when choosing food

Q3-2 I can choose food according to my own nutritional status (fat free, less sugar, etc.)

Q3-3 I’m willing to spend extra time or money on healthy meals
